# Experience of Acceptance and Commitment Therapy for those with mild traumatic brain injury (ACTion mTBI): A qualitative descriptive study

**DOI:** 10.1371/journal.pone.0312940

**Published:** 2025-01-30

**Authors:** Josh W. Faulkner, Jason Chua, Amabelle Voice-Powell, Deborah L. Snell, Maree Roche, John Moffat, Suzanne Barker-Collo, Alice Theadom

**Affiliations:** 1 Te Herenga Waka - Victoria University of Wellington, Wellington, New Zealand; 2 TBI Network, Auckland University of Technology, Northcote, Auckland, New Zealand; 3 University of Otago, Christchurch, Christchurch, New Zealand; 4 School of Management, Fellow NZ Psychological Society, Auckland University, Auckland, New Zealand; 5 School of Psychology, University of Auckland, Auckland CBD, Auckland, New Zealand; University of South Florida, UNITED STATES OF AMERICA

## Abstract

Psychological interventions may make a valuable contribution to recovery following a mild traumatic brain injury (mTBI) and have been advocated for in treatment consensus guidelines. Acceptance and Commitment Therapy (ACT) is a more recently developed therapeutic option that may offer an effective approach. Consequently, we developed ACTion mTBI, a 5-session ACT-informed intervention protocol. To establish the feasibility of this intervention, we wanted to understand participants’ experiences of ACTion mTBI, determine acceptability and identify any refinements needed to inform a full-scale effectiveness trial. We recruited adults (≥16 years of age) diagnosed with mTBI who were engaged in community-based multidisciplinary rehabilitation. After completing the ACTion mTBI sessions, 23/27 (85.2%) participants (mean time post-injury: 28.0 weeks) completed a semi-structured interview about their experience of the intervention. Interviews were audio-recorded, transcribed verbatim and analysed using a qualitative description approach. There were two overarching themes 1) *attacking the concussion from a different direction* and 2) *positive impact on recovery* which depicted participants’ overall experiences of the intervention. Within these overarching themes, our analysis also identified two subthemes: 1) *helpful aspects of the intervention* which included education and ACT processes (i.e., being present and being able to step back) and 2) “*contextual factors that enabled intervention effectiveness*” which included being equipped with tools, cultural and spiritual responsiveness, the therapeutic connection, and the intervention having a structured yet flexible approach to order of delivery to meet individual needs. Participants’ experiences support acceptability, cultural and spiritual responsibility of ACTion mTBI. Suggested refinements included enabling access to intervention over time, not just at one point during recovery and the addition of a brief check-in follow-up.

## Introduction

A mild traumatic brain injury (mTBI) occurs when there is a plausible external mechanism of injury (e.g. hitting the head against an object, vehicle accident, fall or assault) and evidence of an altered mental status (such as loss of consciousness, confusion, or disorientation or not being able to remember what happened) [1]. There is now international consensus that a concussion is considered a form of mTBI [2]. The overall incidence of mTBI is estimated to be 302 per 100,000 people and is highest in males and those that are 16–20 years [3]. Following mTBI, individuals may experience physical (i.e., fatigue, headaches), cognitive (i.e., concentration difficulties) and affective (i.e., irritability) symptoms, commonly referred to as post-concussion symptoms (PCS). PCS can be disabling, impacting many facets of an individual’s life [4–6]. This includes difficulties completing everyday tasks, productivity at work/study and participation in the community [7, 8].

For a considerable percentage of individuals (≈30%) symptoms can persist for months or even years following injury [9]. Psychological factors, when considered alongside demographic variables, the severity of acute symptoms, and clinical characteristics of the brain injury, are among the most robust predictors of unfavourable recovery from mTBI [10–14]. As a result, bio-psycho-socio-ecological prognostic models have been proposed to conceptualise mTBI recovery [15]. In short, these models highlight the role of pre-injury vulnerabilities, early physiological effects induced by the mTBI, and over time, an increasing role for other factors, such as psychological reactions, psychosocial stressors, coping strategies, and medicolegal issues [16, 17]. Consequently, mental health treatment, such as psychological therapy, is recommended within clinical practice guidelines for mTBI [18–20].

A common theoretical approach to psychological therapy is Cognitive Behavioural Therapy (CBT). CBT has gone through several different eras, generations, or waves [21]. The first generation is behavioural therapy which focuses on problematic behaviour and emotion based on conditioning and behavioural principles [21, 22]. The second generation, focuses on modifying unhelpful thought patterns impacting emotions and behaviour, training adaptive thinking and engaging in enjoyable activities using various techniques (e.g., activity scheduling) [23, 24]. The third-wave is based on the concept of context and focuses more on the person’s relationship with thoughts and emotions than on the content of cognitions, emphasizing processes such as acceptance, interoceptive exposure, mindfulness, and values [25, 26].

One such third-wave therapy is Acceptance and Commitment Therapy (ACT) [27, 28]. ACT is based on Relational Frame Theory (RFT) with philosophical roots in functional contextualism [29]. RFT posits that human language and cognition are bound into the ability to identify and generate relational links symbolically between stimuli; and are regulated by the relational context and the functional context of the behaviour [28]. The focus of change within ACT is in the context of the distress rather than the content. ACT is a transdiagnostic psychological intervention that aims to target the psychological process of psychological flexibility [30]. Psychological flexibility is defined as remaining in the present moment, staying open to experience whatever thoughts and feelings show up (good or bad), and taking action in service of values [31]. Following a scoping review of the literature, Cherry and colleagues [32] proposed that the essential components of psychological flexibility are: a) *handling interference or distress* (e.g., receiving new environmental information, experiencing emotional discomfort, facing setbacks); b) *taking action* to manage interference or distress (e.g., engaging in an emotion regulation strategy, persisting, accepting, tolerating, shifting behaviour); and c) taking action occurs in a manner that fits situational demands and *facilitates the pursuit of personal goals or values*.

ACT aims to increase psychological flexibility by utilising six core therapeutic processes: acceptance (the active and aware embrace of private events without unnecessary attempts to change their frequency or form), cognitive defusion (changing the way one interacts with or relates to thoughts), self as context (the part of oneself that is able to step back and watch what is happening), being present (ongoing non-judgemental contact with psychological and environmental events as they occur), values (ways of describing the ways in which we want to live) and committed action (patterns of effective action linked to chosen values) [28, 33]. ACT has a well-established evidence base for improving functioning and well-being in a variety of populations with psychological difficulties and/or medical problems [34–37]. Emerging evidence has indicated that this therapeutic approach may be beneficial in individuals following acquired brain injury (any type of brain damage that occurs after birth) [38–41]. For example, Sander and colleagues [38] conducted a randomized control trial where ninety-three participants with complicated mild (a mTBI associated with brain bleeding, swelling or fractures) to severe TBI, were randomly allocated to eight weeks of ACT or a single session of needs assessment, brief counselling/education and referral. The ACT group showed a significantly greater reduction in psychological distress and improvements in psychological flexibility. The authors concluded that this study provided evidence regarding the beneficial effects and feasibility of ACT for the treatment of psychological distress in persons with complicated TBI. However an attention-matched control group would have better supported these conclusions, and it is unclear if those with differing TBI severities responded differently.

At present, the efficacy of ACT has been examined in severe TBI or mixed acquired brain injury (ABI) samples and has not been explored in relation to mild TBI symptoms or broader recovery from injury. mTBI presents with its own unique characteristics and prognosis and therefore it is important to investigate this therapeutic approach in this population. In regards to this, evidence is currently limited to case studies [42] or within restricted sub-populations (i.e., veterans, -individuals who have served in the military, naval, or air service) [43]. Although these studies provide initial promising results for the benefits of ACT in mTBI, there is a need to examine the effectiveness of this approach in a broader community sample of individuals who have experienced mTBI including uncomplicated mTBI (a mTBI not associated with brain bleeding, swelling or fractures). This is particularly relevant given the unique challenges this group face in terms of the invisibility of the injury, the pressure to return to work and managing expectations of a quick recovery [44–46]. Additionally, in many mTBI rehabilitation settings, only a limited number of psychological services are funded and available. For example, in New Zealand, only 5 hours of psychological services are funded by the national Accident Compensation Corporation (a no-fault insurance provider). Consequently, to aid in the implementation of an ACT approach into clinical practice, there is also a need for ACT to be fit for context and re-designed and tested within a shorter intervention timeframe. As a result, we developed ACTion mTBI, a 5-session treatment protocol that aims to apply the ACT model to improve recovery after mTBI.

Before establishing the efficacy ACTion mTBI feasibility of delivering this intervention protocol within a multi-disciplinary concussion service needs to be established. A feasibility study is a preliminary study to assess the practicality, viability and potential challenges of a proposed clinical trial. The objective of the current study was to understand participants’ experiences of ACTion mTBI and to identify ways to improve the intervention to further inform a full-scale effectiveness trial. Furthermore, this study provides an opportunity for a more informed and nuanced insight into the therapeutic approach, overall intervention and clinical material from participants. This is particularly pertinent for this population in which post-concussion symptoms (i.e., cognitive difficulties, fatigue, headache) could impact engagement and participation in ACTion mTBI. Furthermore, if ACTion mTBI is found to be effective for mTBI, this approach provides an opportunity to examine what aspects of the intervention enabled this to occur. We are particularly interested in understanding whether participants describe ACT processes as being helpful or whether the benefits are produced by more generic ingredients within psychotherapy (i.e. psychoeducation, therapeutic relationship, and emotional disclosure).

## Method

The feasibility trial was prospectively registered with the Australian and New Zealand Clinical Trials Registry (ACTRN12621000594820). This manuscript presents the qualitative data obtained as part of the ACTion mTBI feasibility trial delivered within a multidisciplinary community-based mTBI rehabilitation service.

### Participants and feasibility trial procedures

All participants for the feasibility trial were recruited through Concussion Services in New Zealand. All individuals referred to these services have a confirmed concussion diagnosis by a medical practitioner, receive occupational therapy and physiotherapy input, and may also have had a neuropsychological screening assessment or a specialist medical review. Recruitment took place between August 2021 and June 2023. However, the study was paused between January 2022 and August 2022 because of the COVID-19 pandemic and the periods of government-imposed lockdowns during this time.

Individuals were informed about the study by a clinician within the concussion rehabilitation service. To be eligible for the trial, participants were required to: (1) have a medical diagnosis of mTBI, (2) be aged 16 years or older, (3) be engaged in a multidisciplinary concussion rehabilitation service, (4) have elevated levels of post-concussion symptoms as indicated by a score on the Rivermead Post-Concussion Questionnaire of 16 or greater [47], and (5) have an absence of any pre-existing major neurological condition (e.g. Epilepsy). For this analysis participants also needed to have received at least one session of the ACTion mTBI.

### ACTion-mTBI intervention

A concise overview of ACTion mTBI is provided below, further detail according to the TIDieR checklist is available in the supplementary materials. ACTion mTBI aims to address mTBI-related needs and promote recovery. Due to the time-limited nature of the intervention, the development of ACTion mTBI was informed by Focused ACT [48]. The treatment protocol was designed in consultation with a trained facilitator and expert in ACT with extensive experience in mTBI (JM). ACTion mTBI consists of 5 sessions, with each being approximately 50 minutes in duration. ACTion mTBI incorporates all six processes of the ACT model including contact with the present moment, identifying and working with personal values, facilitating acceptance, defusion techniques, observing thoughts and feelings and identifying action. ACTion mTBI adapts experiential exercises, metaphors, discussion and homework within an mTBI context which aims to: (a) cultivate awareness and acceptance of thoughts and emotions about mTBI, (b) recognize the impact that rigid thoughts/beliefs, behaviours, and responses to emotions have on mTBI recovery, and create a more flexible response to such experiences; and (c) clarify personal values and commit to pursuing meaningful activities aligned with these values whilst recovering from mTBI.

A recent report of the Association for Contextual Behavioural Science (ACBS) task force of contextual behavioural science research stated: “It is unhelpful to allow applied psychological science to remain at the level of extensive intervention protocols, when the spirit of idiographic functional analysis linked to processes of change requires a more personalized approach” ([49] pp. 176). Consequently, ACTion mTBI was delivered with flexibility based on clinical judgment in accordance with the participant’s presentation. For example, if a central feature of a client’s presentation was severe fusion with unhelpful cognitions driving the presenting issues, then components within session 3 ‘unhooking from the mind’ may be delivered earlier in the intervention. What is critical in the delivery of ACTion mTBI is that the therapist adopts an ACT stance [50] and that all processes of the ACT hexaflex are covered within the five sessions. The ACTion mTBI protocol was delivered by JF a board registered Clinical Psychologist and Neuropsychologist with 8 years of clinical experience working with mTBI who has advanced-level training in ACT. He had ongoing supervision by an ACT-trained supervisor when delivering the intervention.

### Qualitative interviews

Following completion of ACTion mTBI and follow-up questionnaires, all participants receiving at least one session of the ACT intervention were invited to engage in a semi-structured interview to explore their experience of the intervention. Semi-structured interviews were conducted by a Research Assistant or a Research Fellow (JC) independent of the research team (see interview guide in Table 1). The interview guide was based on a previously used schedule evaluating a psychological treatment in individuals with TBI [51].

**Table 1:** Interview guide.

Questions
• How did you find the treatment you received?
• Did the treatment make sense to you?
• What did you like about it?
• Was there anything that wasn’t helpful or could be improved?
• Was the treatment responsive to your culture?
• How do you feel about managing your recovery now you have finished treatment?
• What would you say about this treatment to someone else who has experienced a brain injury?

Participation in the interview was optional and separate informed consent was obtained. Interviews lasted between 15 and 60 minutes. All interviews were conducted over Zoom or Teams teleconference platforms, audio-recorded and transcribed verbatim. All identifying features were removed from the transcriptions to ensure anonymity before analysis.

### Qualitative analysis approach

As person’s recovery from mTBI can be influenced by a range of injury related, contextual and psychosocial factors, such as the person’s sense of self, social networks, role in society and life demands, social constructivism underpinned the study. The qualitative description approach [52] was used to analyse the data as it aims to improve understanding of a phenomenon, from the perspectives and worldviews of the people involved enabling cultural responsiveness of the intervention to be explored [53]. Qualitative description aligns well with a mixed method approach [54], striving to provide an in-depth description of an experience without moving too far away from the data [55, 56]. This approach maintains the integrity of participants perspective and use of language and aligns well with the objectives of this study which is to understand participants experience and perception of ACTion-mTBI. As the aim was to stay close to the data and describe participant experiences, a descriptive approach was felt to be most appropriate.

All transcripts were independently coded by two researchers (AT and A-VP) using a data driven approach (i.e., there were no predetermined codes used to guide the data analysis). The researchers familiarised themselves with the data by reading the transcripts in full, noting the overarching story and key issues identified by the participant and then coding sections of text to summarise the participant’s experience and the meanings participants attributed to it [52]. Nvivo software was used to facilitate analysis. Preliminary codes were discussed, refined and combined to generate a final code list. Any differences in interpretation or where there was uncertainty around coding were discussed with the wider research team until a consensus decision was achieved. A description of each code, and codes combined within it, were used to record the analysis process. Extracts coded within each code were then reviewed by the team (AT, A-VP, JC and JF) to refine the code name to ensure it accurately reflected the data within it. Codes aimed to capture both positive and negative aspects of the intervention experience.

In accordance with the qualitative description approach processes were implemented to ensure credibility, confirmability, dependability and transferability as outlined by Bradshaw et al, [57]. For example, both the interviewers and coders kept reflexive notes. An audit trail was kept to defend decisions made during the research process, participant quotes are integrated into the findings to ensure robustness of the analysis approach [58]. Additionally, a second reviewer with limited knowledge of ACT and independent to the trial research team was involved in the coding and analysis of transcripts to prevent bias and ensure trustworthiness [58]. To provide context for each quote while protecting anonymity, extracts from the interviews are presented with information on the participants’ gender and time since injury.

## Results

Twenty-three of the twenty-seven participants assigned to the ACT intervention completed an interview (85.2%). The participants ranged in age from 20 to 65, with a mean age of 33.83 (SD = 12.99) years. As seen in Table 1, most of the participants were female, were predominately New Zealand European and were generally well educated. Participants were on average about 7 months post-injury; the most common mechanism of injury was a fall, followed by hit by an object. About half of participants had previously experienced a mTBI and many had a pre-injury mental health diagnosis (see Table 2).

**Table 2:** Demographic and injury characteristics of participants.

**Demographics**		
	**Mean**	**SD**
*Age*	33.83	12.99
	**N**	**%**
*Gender*		
Female	21	91.3%
*Ethnicity*		
Māori	3	13.0%
NZ European	14	60.9%
Other	6	26.1%
*Education History*		
High school or less	5	21.7%
University/Tertiary	18	78.3%
*Previous concussion history*		
Yes	11	47.8%
No	12	52.2%
*Mental health history*		
Yes	14	60.9%
No	9	39.1%
**Injury Characteristics**		
	**Mean**	**SD**
*Time since injury* (weeks)		
Pre-treatment	28.0 [7–134]	28.1
	**N**	**%**
*Mechanism of Injury*		
Motor Vehicle Accident	3	13.0%
Fall	9	39.1%
Assault	3	13.0%
Hit by object	8	34.8%
*Other Injuries sustained*		
Yes	14	60.9%
No	9	39.1%

**Fig 1:**
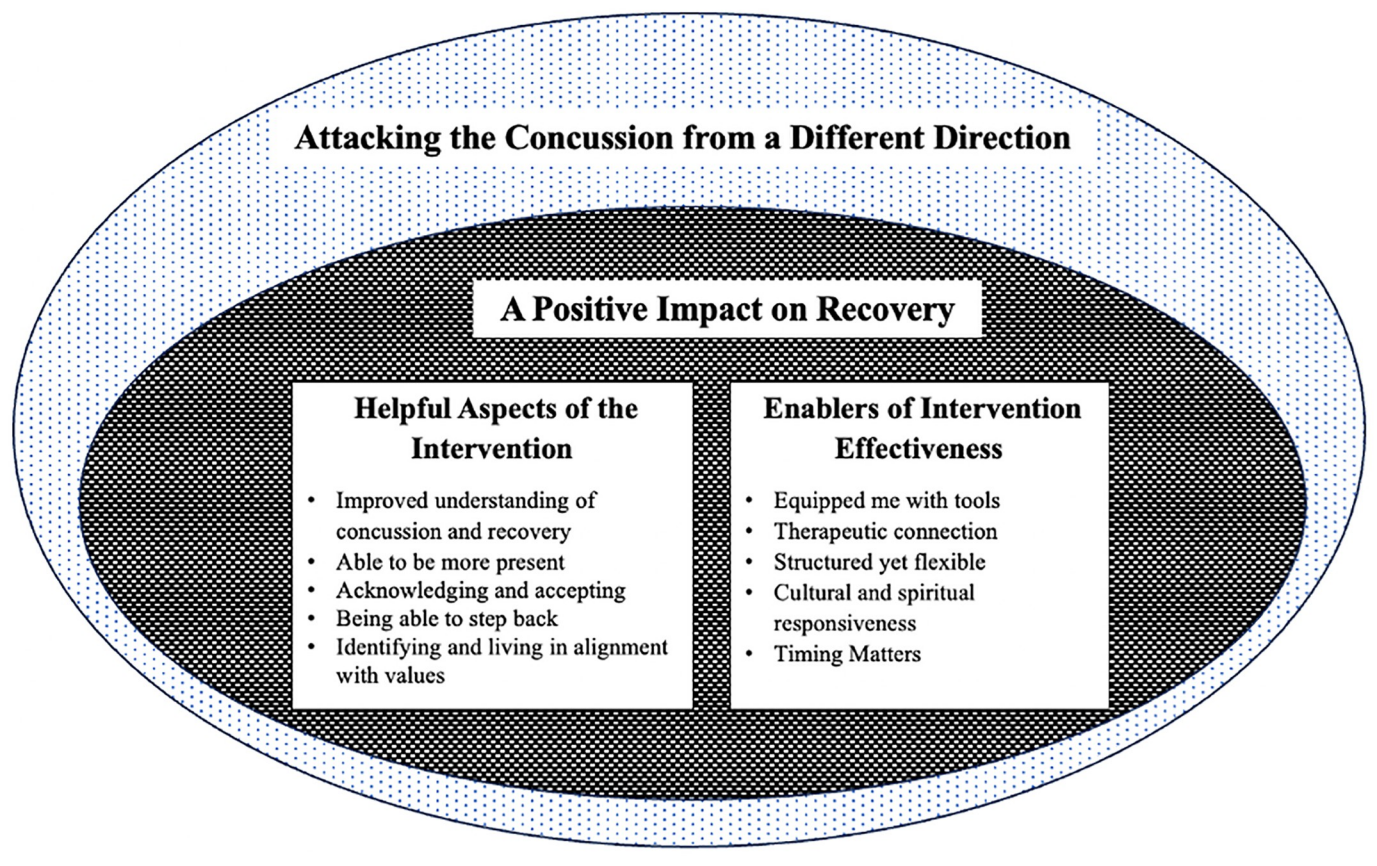
Schematic representation of the four themes.

Participants’ experiences and reactions to ACTion mTBI were reflected by two overarching and two sub-themes, as illustrated in Fig 1. The first theme, *Attacking the concussion from a different direction*, reflected participants’ overall experience of ACTion mTBI. A *positive impact on recovery* captured the impacts that this intervention was perceived to have had on their mTBI recovery. Within this, our analysis was able to dissect how *attacking the concussion from a different direction* had *a positive impact on mTBI recovery*. This occurred through the two sub-themes *helpful aspects of the intervention* and *enablers of intervention effectiveness*.

### Overarching themes

#### Theme 1: Attacking the concussion from a different direction

Participants talked about initially being sceptical of the therapy but went on to say *“I’m surprised how good I found it”* (female, 86 weeks post-injury) and “*it was really good*. *I wasn’t expecting it to be good* (female, 29 weeks post-injury). The participants all described that they found the intervention helpful in some way in managing their recovery.

“*I found this possibly the most helpful course of treatment for my concussion*. *I’ve done a fair few of them*, *but this has been the most helpful*, *tangibly helpful*.

*(female*, *134 weeks post-injury)*.*”*

When asked about what they liked about ACTion mTBI many participants described that the treatment targeted a different aspect of their mTBI and gave them a new approach to manage it.

*“I would describe the treatment… kind of attacking the concussion from a different direction*, *so it’s more the emotional side of it*.. *and also like tools to actually get ahead of your concussion and get some distance from it because it kind of takes over your life*

*(female*, *27 weeks post-injury)*.*”*

Participants talked about how ACTion mTBI addressed more than the physical aspects of mTBI and stressed the importance of this. Participants talked about needing to deal with the trauma, changed perceptions of self and the challenges they had coping with the psychological impacts of not being able to do things the way they used to. Some participants were scared and concerned that they had not recovered from their injury. ACTion mTBI enabled them to address the emotional and cognitive aspects of concussion recovery that they were aware of but had felt lost in being able to address.

*“I think it’s incredibly vital*. *Um*. *Just incredibly vital*. *Just to take a different approach*. *I think a lot of the concussion stuff has been focused on the physical effects*, *but a concussion is not entirely physical*. *It’s mental as well*. *I’ve had a lot of people go*. *Your recovery is taking longer because I have existing mental health problems*. *Which is a very frustrating thing*, *because then no one gave me any tools to solve it … This gave me the tools to start solving it for myself*. *And when you feel*, *in my experience when I was concussed*, *I felt incredibly powerless*. *I didn’t have*. *There’s not a lot you can do*, *they just have to tell you to sit in a darkroom for the next few months*. *But this gives you some tools you can do*.*”*

*(female*, *25 weeks post-injury)*

Many talked about the difference between Action mTBI and other therapies that they had previously received and appreciated the different approach of Action mTBI.

*“I’ve never had good experiences of therapy in the past*. *They [past therapy courses] were usually focused on (like) CBT or similar (um)—or DBT—similar (sort of) behavioural therapies*. *And I found this one was far more—like it really suited me a lot better*. *(Um*, *kind of like) the idea of accepting things rather than trying to change them*.

*(male*, *22 weeks post-injury)*.*”*

#### Theme 2: A positive impact on recovery

Participants described feeling empowered as a result of ACTion mTBI, with all participants describing how the intervention had a positive impact on their recovery. Some participants felt that they had recovered by the end of the intervention.

*“Yeah*. *I think realising that all of my … concussion symptoms of headaches and pain*, *realising that it was a*, *that all my emotions were driving it*, *like feeding into it*. *And once [the therapist] was able to provide those techniques*. *Yeah*. *I don’t know*, *it just*, *it kind of just eased up and then went away*

*(female*, *10 weeks post-injury)*.*”*

Others reported that they had not fully recovered by the end of the intervention but that they believed ACTion mTBI helped to reduce symptom experience to a more manageable level and increased their ability to engage in everyday activities. Additionally, some participants described feeling that they had plateaued or felt stuck in their recovery journey before they engaged in ACTion mTBI. This gave participants a sense of hope for the future.

*“About six months ago*, *I was probably at the lowest I have ever been*. *And I have tried a lot of things to recover from my concussion… And my concussion was beginning to define me*. *Um and I don’t*, *I feel that I am more in control now… So that certainly gave me tools to let’s just say*, *turn on a light or see the light at the end of the tunnel*, *because I certainly probably up until that point could not see any significant end to this*.

*(*female, 86 weeks post-injury*)*

Participants also reflected that ACTion mTBI helped them with other aspects of their mental health, their overall life, as well as their concussion recovery. The intervention addressed issues that were not directly related to the concussion but were of importance to the person and affecting their recovery (e.g., cultural identity issues, mental health concerns).

*“I struggled also a lot with anxiety and depression and couldn’t also cope*. *Well*, *that also helped bring light to those*. *And they are just basically non-existent*. *Yes*. *I still have bouts of them*. *But I can deal with them more than I was able to*

*(female*, *38 weeks post-injury)”*

Participants who had not fully recovered described feeling more confident and in control of their recovery journey following the ACTion mTBI.

*“it doesn’t feel like a tsunami anymore*, *it feels more like just a wave*. *And you’re like*, *you know*, *you’re at the beach*, *and you’re just jumping over the wave and you’re like*, *you know*, *getting splashed within*, *that’s fine*. *But then the calm water comes*, *and*, *you know*, *there’s gonna be another wave*, *but you’re totally okay*. *Instead of feeling like*, *Oh*, *my God*, *everything’s just gonna pile up*, *and I’m just gonna be like*, *taken away and this tide*, *and that’s that I’ve done and*, *you know*, *can’t move forward here*. *So I definitely feel like the difference between having the treatment in the way that I view my recovery and how I deal with the things that come up with my recovery*

*(female*, *20 weeks post-injury)*.*”*

### Subthemes

#### 1. Helpful aspects of the intervention

When asked about what particular parts of the intervention they liked or found particularly helpful, the following areas were identified.

*Improved understanding of concussion and recovery*. Participants expressed that a key component of the intervention was that they had received clear explanations about their concussion and why they felt the way they did. This was delivered using language and terms that were understandable. This helped participants understand their symptoms in a new way, reducing confusion and uncertainty.

*“Um well [the therapist] kind of explained what concussion was because I had no clue and [the therapist] explained what happened to me which no one could explain to me so like that was amazing to hear because I had been to like doctors and hospitals and no one could explain to me what was going on and I was just so confused*, *like what had happened to me…*

*(female*, *134 weeks post-injury)”*

This improved understanding of mTBI and why symptoms were persisting, helped set participants up to understand and engage in ACTion mTBI.

*“So you have*, *you know*, *that explanation*, *and understand it’s your brain*, *you haven’t made this up… And then the ongoing sessions to kind of understand*, *you know*, *what are you doing*, *and the behaviours that might be tripping you up*, *and the ways in which you engage with that*. *And yeah*, *it was brilliant*, *very well explained…*

*(female*, *8 weeks post-injury)*.*”*

*Able to be more present*. Participants talked a lot about how ACTion mTBI helped them to understand how they were feeling following the mTBI and the techniques that assisted in helping them to feel more like their usual selves.

*“it was definitely life changing wealth like from my perspective it was like it was*, *it really did help me to become present*, *and be there with my family which is amazing because I had no idea what was happening like*, *saying to the doctors like I’m here but I’m not here*, *no one knew that what that was*, *like she’s weird*, *but then all that took was to be present*.

*(female*, *134 weeks post-injury)*.*”*

The experiential techniques were mentioned by participants as being particularly useful to help bring them back to the present moment when they were overwhelmed.

*“That first technique was that [the therapist] used anchoring… It helped ground me because I get so caught up in my head*, *and you’re like*, *hey*, *you’re not even breathing properly*, *like deep breaths*, *you know*, *and it’s more than just deeper breaths though it’s realising its bringing me*, *it brought me back into my present*, *like*, *I’m worrying about all these 10 million things that and my past or my future*, *and I’m not allowing myself to think about what’s going on around me*.*”*

*(female*, *10 weeks post-injury)*

*Acknowledging and accepting*. Acknowledging and accepting the thoughts and feelings that were evoked following a mTBI was felt to be a valuable part of ACTion mTBI. Participants talked about how they had previously been deflecting away from these experiences and the impact this was having on their recovery.

*“I’m a massive deflector of emotions*. *I don’t feel emotions*, *but that was actually draining me a lot cause turns out a lot of energy goes into that sort of thing*, *so a lot of our treatments sort of focused on how to sort of work with emotions and feelings and how to sit with them*, *so I expended less energy pushing them away and trying to get through*. *Yeah*, *and that would help me get out of the patterns I’d fallen into*

*(female*, *25 weeks post-injury)*.

Participants talked about the benefits of acknowledging their emotions and giving themselves permission to feel these. It was described as freeing and allowed participants to feel more in control of their emotions, whereas previously they had felt overwhelming and unmanageable.

*“[the therapist] called it the dropping anchor*. *Because I’d gotten really*, *really upset… and it was all very much like a revelation to me*, *it was like*, *Oh*, *I can have these big feelings*. *And they can still exist*, *and I don’t have to be terrified of them*. *And*, *okay*, *now that I’ve done that*, *I know that I can*, *so I will try to do that myself… So you know*, *that was that was brilliant*

*(female*, *8 weeks post-injury)*

*Being able to step back*. Participants discussed how ACTion mTBI equipped them to distance themselves from thoughts that were previously dominating and overwhelming them.

*“when we were examining … it meant that we went back into (like) my past and (like) where I got those thoughts from*. *And imagining those thoughts as someone else*, *if that makes sense*. *I thought–so initially [the therapist] said to imagine the thoughts as another person saying them*. *And [the therapist] said*, *“Oh*, *some people say it’s a troll or a*, *(you know)*, *evil man or something like that”*. *And while I initially thought*, *‘that sounds ridiculous’*, *it did help to detach those thoughts from myself*

*(female*, *22 weeks post-injury)*.

Participants discussed how experiential techniques allowed them to acknowledge their thoughts but to be no longer driven by them. This supported participants to see their thoughts as less threatening, regain a sense of control when they occurred, and prevent self-perpetuating cycles from occurring.

*“Um so another one [the therapist] gave me …you can sort of imagine that’s*, *um*, *so it’s like there’s a part of your brain which is the protector and that’s kind of the part that can be overprotective*. *So um yeah*, *you can sort of say*, *oh these thoughts about oh I shouldn’t go out and do this thing because it might be scary or I might have*, *might be awkward you can sort of say*, *oh that’s what the protector trying to try to protect me by saying giving me these warnings but I don’t necessarily need to follow all of that advice*. *I can sort of say hey protector like thanks for having my back but you’re a*, *yeah you don’t need to be quite so protective*, *so yeah that was a*, *that was useful so yeah it was kind of saying you don’t need to listen so much to the protector …*

*(male*, *16 weeks post-injury)”*

### Identifying and living in alignment with values (Values and committed action)

Action mTBI aimed to identify and connect people to live a life and to respond to situations in a way that was congruent with their values. Many participants found this a helpful component of the intervention assisting them to decide where to invest their time and energy by prioritising what’s important.

*“So we talked about making choices that go towards your values um instead of choices that go away from so you know like the um in part of their unhooking from those kind of horrible thoughts or the disturbing ones … It’s important to me to be functional and healthy and able to do what I need to do for my family*, *yeah but I realised that a lot of my choices around my health and wellbeing were away moves because I’d prioritise the other stuff over top and so that my health and wellbeing wasn’t that flash*

*(female*, *6 weeks post-injury)*.*”*

The focus on identifying values was felt to be a strong way of integrating participants’ cultural and spiritual beliefs into the therapy.

*“… one was actually going kind of deep diving more into what my actual values are*, *I guess*. *And that’s when [the therapist] created the whole whare* [Māori translation: house, building, residence, dwelling, shed, hut, habitation] *and incorporated my culture*. *So coming up with the* pou [Māori translation: erect, establish, fix, elevate on poles] *…so you know how you have a house*. *And it has like*, *the things that keeps the house up*. *Those are the pou*. *So the support the things that are supporting the roof and*, *and everything to stay up*. *So those were my values*, *and [the therapist] was able to incorporate that*, *the cultural aspect into the techniques that [the therapist] had been using with me*

*(female*, *10 weeks post-injury)*.*”*

#### 2. Enablers of intervention effectiveness

There were a number of contextual factors identified by participants that facilitated their engagement and the effectiveness of ACTion mTBI.

### Equipped me with tools

All participants commented that they liked how Action mTBI gave them a number of different practical tools they could use to assist them in their recovery. Participants appreciated that these tools were practised in session with the therapist and they could apply them flexibly which enabled participants to incorporate the techniques into their context.

*“I’m very grateful that we focused a lot on tools*. *So it was sort of [the therapist] would help me sort of get to a point where I would recognise things and then go OK*, *here’s some actions we can do*. *And they were things that I could really easily slip into my routine*, *things that I’m still doing now”*.

*(female*, *25 weeks post-injury)*

### Therapeutic connection

Participants talked about how the therapist made them feel during the sessions was central to their engagement in the intervention. The therapist was described as holding a space for them, enabling them to take control of their own recovery which gave them hope. Participants highlighted the importance of having someone who was approachable that they could talk to, but also to keep them on track during the intervention. This created a space for participants where they could talk freely and not feel judged.

*“um [the therapist] just really held the light of hope I think for me*, *um I did feel like there was no hope and there’s no help for me but [the therapist] just held it … and really just building me up and letting me know you are in the driver seat you are in control and you may not feel that way but I’m gonna hold onto that hope and hold onto that light for you for when you’re ready to take it*

*(female*, *16 weeks post-injury)*.*”*

### Structured yet flexible

Participants liked the structure and practical nature of the sessions.

*“… the practicality of the sessions were*, *was just what I needed it*. *I think it’s just what anybody with a concussion or brain injury needs is that clarity*, *um structure*, *and practical tools because everything else can seem a little bit wiffly-waffly*

*(female*, *26 weeks post-injury)*.*”*

They also felt that whilst the intervention was structured it was able to be flexible enough to respond to what they needed particularly if events were coming up, as well as reflecting on how things went since the last session. The recaps were felt to be a useful component of the intervention. Some participants described that knowing that the treatment time was limited helped them to remain focused to ensure that they got the most out of it. For others, the time-limited approach did initially create some reservations and pressure. Some participants did state they would have appreciated further sessions to really grasp some of the concepts raised. If more sessions were not available participants suggested that a short ‘check-in’ could be offered.

*“… you’re aware right from the beginning that there’s only five sessions so your thoughts at that point go to … I don’t know what [the therapist] can accomplish in these five weeks*, *so you’re very aware that you want to get in the way of that and hold you know like everything that needs to be done in that five weeks…*. *I was always aware*, *I think five was probably enough but yeah…I could have easily had another one just to review but you know it all works out because you can do that on your own*, *just take the time to do it*

*(female*, *12 weeks post-injury)”*

### Cultural and spiritual responsiveness

Participants reported that ACTion mTBI was responsive to their culture and spirituality and because of this it created a sense of safety as they engaged in the intervention.

*“I think it was one of my values I said that spirituality… And it was all around basically my head because in Māori culture*, *the head is very sacred*. *And [the therapist] was able to*, *in a way*, *make it feel Yes*, *it’s sacred*. *We can work in a way to keep it like that without touching too much*, *but also heal it in the process was*, *yeah*, *it was really good*.

*(female*, *31 weeks post-injury)*.*”*

Participants expressed appreciation for how culture was addressed in ACTion mTBI. Participants liked that the therapist made sure to inquire and made sure to check in before making any assumptions. This meant that the integration of culture into the intervention aligned with the participant’s worldview.

*“it was really good that [the therapist] wasn’t … I don’t know*, *the word for it*. *But people can be quite yeah this is what your culture is*. *And that’s that*, *and this is*, *so this is how I’ve laid out our programme [the therapist] wasn’t like that*, *from the very beginning*. *[the therapist] was like*, *Hey*, *I’d like to do this*, *would you like to do this*, *you know*, *continuously validating and consenting to the cultural aspect*, *and just acknowledging …*. *Yeah*, *and I think their continuous validation*, *opening space and consent helped*. *So it allowed me to kind of give my points of view on top of my experience of my culture…*

*(female*, *10 weeks post-injury)”*

### Timing matters

The timing of the intervention was felt to be important to engagement. Some participants talked about how they wished that they had been access to ACTion mTBI sooner.

*“I wish I’d had access to this kind of support a lot earlier…It was very late in the process… And I think that earlier it just would have probably helped lift my trajectory*, *my recovery trajectory a lot sooner*

*(female*, *25 weeks post-injury)”*

However, another participant who had their injury three months previously, reported that they might not have been ready for the intervention if they had been offered it sooner in their recovery.

*“I would say very positive and worth doing*. *Um I think the timing of it was quite important in terms of then again my OT flagged at the right time; I wouldn’t have found it useful right at the start of my journey*, *I think my concussion was six months ago that’s when the support’s come in useful*, *where it’s become more of a long term thing*

*(female*, *20 weeks post-injury)”*

## Discussion

This qualitative description study sought to understand the experiences of individuals participating in ACTion mTBI, a time limited ACT intervention for mTBI, within a community-based multidisciplinary rehabilitation service. It aimed to identify possible impacts of the intervention, understand what aspects of the intervention drive these impacts, and identify any required modifications or unexpected outcomes. During the interviews, participants reflected on the psychological difficulties that they experienced after their mTBI. This included heightened distress, mood changes, uncertainty, anxiety, and changes to self-concept. These experiences are consistent with the well-known mental health difficulties that can occur after mTBI [59, 60]. These mental health difficulties can reflect pathological consequences of the injury, environmental and post-injury effects and/or continuation/exacerbation of pre-injury mental health conditions [13, 17]. Our qualitative description analysis revealed that ACTion mTBI was able to identify and address these psychological difficulties as indicated by the overarching theme: *attacking the concussion from a different direction*. Participants noted that ACTion mTBI went “beyond the physical” and targeted a different aspect of their mTBI recovery, the emotional and cognitive effects of the injury. Interestingly, participants were often aware of the importance of this but felt powerless or overwhelmed in their ability to address or manage these aspects of their well-being prior to the intervention. This illustrates the importance that psychological interventions can have in mTBI recovery and are in accordance with consensus guidelines for mTBI rehabilitation [18–20].

Participants described how ACTion mTBI *attacking the concussion from a different direction* was beneficial as reflected in *Theme 2*: *a positive impact on recovery*. These benefits were diverse. Some participants described ACTion mTBI as contributing to complete recovery, whilst others described alleviation in symptom severity and improved daily functioning. Importantly, no adverse reactions to the intervention were reported by participants. These results are promising and suggest that ACT may be a useful psychotherapeutic modality for supporting recovery after mTBI. This finding is consistent with a limited, but growing evidence to support the use of ACT for more severe TBI and stroke populations [38–43], and with more expansive research on the benefits of ACT for a range of health conditions including cancer, chronic pain, insomnia and fibromyalgia [61–66]. In addition, a promising finding was that even if some participants had not felt fully recovered from mTBI, they expressed confidence and self-efficacy in their recovery journey. This aligns with a key objective of ACTion mTBI. With only five sessions available, ACTion mTBI aims to provide participants with a toolbox of techniques that they can draw on to support their ongoing recovery and well-being journey. Additionally, in accordance with the ethos of ACT, the primary objective of this approach is not to reduce, eliminate or control internal experiences [28, 33]. Instead, in accordance with ACT, ACTion TBI aims to cultivate a more flexible response to thoughts and emotions about mTBI and to clarify values and commit to the pursuit of more meaningful activities [33]. It is reassuring that the experience that participants had of ACTion mTBI on their mTBI recovery, aligns with these objectives.

Under theme 3, *Helpful Aspects of the Intervention*, we were able to discern what aspects of the intervention resulted in the positive impact that ACTion mTBI had on mTBI recovery. Of significance, this theme consisted of ACT processes which align with the therapeutic components of the ACT model (commonly referred to as the ACT hexaflex). These included: able to be more present (contacting the present moment), acknowledging and accepting (acceptance), being able to step back (defusion) and identifying and living alignment with values (values and committed action) [33]. This provides evidence that some of the effects that were produced by ACTion mTBI are driven by core ACT processes. It is also worth noting that participants described a range of ACT processes and there did not appear to be a dominant favoring of one process over another. This suggests that ACTion mTBI may strike the balance in how the ACT model is embedded and delivered throughout the intervention. One exception is however noteworthy, which is self as context. Commonly referred to as the “observing self”, self-as-context is the stable perspective from which all self-relevant processes can be seen [67]. Self as context is implicit in several of the core concepts of ACT such as defusion, acceptance and contacting the present moment [68]. Thus, even though not explicitly stated by participants, the fact that these concepts were described by participants illustrates that self as context has been harnessed by ACTion mTBI. However, experts in ACT argue for the importance of self as context in ACT and stress the importance of making it explicit within the therapeutic context [67]. This may be particularly important for mTBI recovery where changes in sense of self can often be a salient driver of psychological distress [69]. These findings suggest a need to review and reflect on how self as context is delivered within ACTion mTBI. To aid in this, future research would benefit from understanding participants’ experience of this aspect of the intervention.

The theme, *Helpful Aspects of the Intervention* also revealed that the positive impact of ACTion mTBI on recovery was also due to the education provided within the intervention. In fact, it was clear from the participants’ experience that this part of the intervention was critical. Participants noted that this enabled them to understand their injury and their recovery experience which reduced feelings of uncertainty, confusion and fear. These feelings were salient for many of our participants. Research illustrates that these factors can significantly contribute to symptom persistence and incomplete recovery after mTBI [44], and as a result, education has been stipulated as being an essential part of mTBI rehabilitation [18]. Given its importance, based on participants’ experience, it is reassuring that the education component within ACTion mTBI is achieving its intended outcome. In addition, participants also described how the education within ACTion mTBI enhanced their engagement and participation in the entire intervention, which likely influenced the benefits produced by the ACT model. Thus, education appears to be an essential ingredient within ACTion mTBI to facilitate participation and enhance the effectiveness of the intervention.

Through theme 4: *Enablers of intervention effectiveness* we were able to unpack the contextual factors that facilitated engagement and the effectiveness of ACTion mTBI. Participants all liked how ACTion mTBI equipped them with techniques that they could incorporate into their context to assist with recovery. Experiential exercises are integral to ACT as they offer a practical and hands-on approach to learning and applying ACT principles and increasing psychological flexibility [27, 31]. Participants resonated with this approach and often expressed appreciation for the practical nature of the intervention. Participants also highlighted the significance of the therapeutic relationship. Within the ACT model, the development of a consistent and collaborative therapeutic relationship, often referred to as the ACT therapeutic stance, is of central importance [70]. The ACT therapeutic stance is open, accepting and non-judgmental [50]. Creating this relationship provides the context to facilitate engagement and ensure the ACT model can be implemented in a way to maximise its effect.

Participants also provided valuable feedback on the structure of ACTion mTBI and when it could be delivered. Interestingly, some participants suggested adding in an additional follow-up session to support the maintenance of the intervention effects. The research evidence strongly supports the addition of booster sessions [71] and should be considered when implementing ACTion mTBI in the future. Further regarding its structure, ACTion mTBI is an intervention protocol and it was useful to know that many participants liked the structure of each session. Importantly though, and in accordance with the ACT model [31], participants also appreciated the flexibility of ACTion mTBI and its ability to address the most salient issues for the client. Adopting this flexibility was also suggested by participants in regard to when the intervention should be delivered (i.e., “timing matters”). Interestingly, many participants suggested that they would have liked to have received ACTion mTBI earlier in their recovery, however, other participants highlighted the need to feel ready to enable active engagement in the intervention. This aligns with treatment guidelines that advise early psychological treatment to address and mitigate the influence that psychological factors can have on recovery [18]. Finally, participants also reported that ACTion mTBI was culturally and spiritually responsive and could therefore adhere to the specific individualistic of each client that engaged in the intervention. This is consistent with a body of evidence demonstrating the applicability and usefulness of ACT in a range of different ethnic and cultural groups [72–75]. In summary, being experiential, developing a strong therapeutic connection, cultural and spiritual responsivity and being flexible in intervention delivery and timing must occur when delivering ACTion mTBI.

The results of this study do need to be considered in the context of its limitations. First, our participant sample was predominately female. There is evidence that females may experience more heightened emotional distress (i.e., anxiety) after mTBI [76]. It may therefore be that this group is in greater or is more open for psychological treatment to address these difficulties. However, males are at a heightened risk of experiencing mTBI [3]. In addition, our sample was also relatively small, were more likely to be highly educated and were undergoing treatment for mTBI. This limits our ability to generalise the findings of this study to other demographics, as well as the wider mTBI population and it will be important to obtain the perceptions of ACTion mTBI from a larger and more diverse cohort. Second, our analysis was conducted on interviews with participants—85.2% of whom completed the intervention. Although we had a high retention of participants in the treatment, those who took part in the interviews may have more positive experiences of the intervention than those who declined. Thirdly, we conducted interviews following the completion of the intervention and we therefore did not collect data on the longer-term effects of ACTion mTBI on mTBI recovery. We cannot assume that the benefits described by participants in this study translate to sustained changes in recovery over time. There is also inherent subjectivity when conducting qualitative research that needs to be acknowledged. Finally, this study interviewed participants of one treating psychologist who delivered ACTion mTBI. Attributes such as therapist style, rapport, communication style and experience unique to the treating psychologist should therefore be kept in mind with interpreting the results. Future research should establish experiences of ACTion mTBI from a more diverse cohort of treating psychologists.

In conclusion, this study highlights the perception and experience of individuals who have engaged in an ACT-informed intervention for mTBI; ACTion mTBI. This analysis was able to determine that ACTion mTBI has a positive impact on recovery and therefore provides preliminary support for the utilisation of this psychotherapeutic modality in mTBI rehabilitation. Importantly, this study was able to provide valuable insights into how this intervention produces these effects and the value that *attacking the concussion from a different direction* can have on mTBI recovery and overall well-being. Based on participant reflections, ACT processes were found to be helpful in addition to generic ingredients within psychotherapy (i.e., psychoeducation, therapeutic relationship, and emotional disclosure).

## Supporting information

S1 Table ACTion mTBI TIDieR checklist. (DOCX)
