# Supplementary material for: Experience of Acceptance and Commitment Therapy for those with mild traumatic brain injury (ACTion mTBI): A qualitative descriptive study
Source: PLoS One. 2025 Jan 30;20(1):e0312940. doi: 10.1371/journal.pone.0312940 (PMC11781712; doi:10.1371/journal.pone.0312940)
Supplement: S1 Table — (DOCX) [file pone.0312940.s001.docx]

Supporting Information

S1 Table 1. ACTion mTBI TIDieR Checklist

| TIDIER item | Description |
| --- | --- |
| 1. **Brief Name**   Provide the name or a phrase that describes the intervention. | Acceptance and Commitment Therapy for Mild Traumatic Brain Injury (ACTion-mTBI) |
| 1. **Why**   Describe any rationale, theory, or goal of the elements essential to the intervention. | Pre- and post-injury psychological factors are amongst the strongest predictors of prolonged recovery following mTBI and treatment of mental health difficulties are stipulated in treatment consensus guidelines. However, evidence for the most commonly used psychological treatment (Cognitive Behavioural Therapy (CBT)) is limited and inconsistent. There is therefore a need to examine the efficacy of other psychological interventions. Acceptance and Commitment Therapy (ACT) has the potential to improve psychological service provision for mTBI. Despite the growing body of evidence in support of ACT across other clinical populations (e.g., chronic pain), the effectiveness of ACT for TBI has only been examined in moderate/severe injuries. There continues to be a paucity of evidence from clinical trials on the effectiveness of ACT for mTBI. In response to this clinical need, we developed ACTion mTBI. ACTion mTBI is, a new manualised ACT protocol for mTBI. The intervention aims to: (a) cultivate awareness and acceptance of thoughts and emotions about mTBI; (b) recognize the impact of thoughts, behaviours, and responses to emotions on mTBI recovery and; (c) clarify personal values and commit to pursuing meaningful activities aligned with these values. |
| 1. **What Materials:**   Describe any physical or informational materials used in the intervention, including those provided to participants or used in intervention delivery or in training of intervention providers. Provide information on where the materials can be accessed (e.g., online appendix, URL). | A therapist manual describes the procedures involved in designing and creating the intervention (contact the first author for further details and access). It provides background information, delivery guidance, tips, and session content. Materials include (1) session content (2) example scripts and (3) therapist tips which provide guidance and suggestions on how to deliver the content within a mTBI context. Materials also include a client workbook which includes hand-outs and worksheets of the intervention content. |
| 1. **What Procedures:**   Describe each of the procedures, activities, and/or processes used in the intervention, including any enabling or support activities. | ACTion mTBI incorporates all six components of the ACT model and adapts experiential exercises, metaphors, discussions and homework to an mTBI context. Sessions focus on dropping the struggle, moving towards values, unhooking from the mind, the noticing self, committed action, and review and relapse prevention. The therapist manual provides detailed information about the procedures employed. |
| 1. **Who provided**   For each category of intervention provider (e.g., psychologist, nursing assistant), describe their expertise, background and any specific training given. | The intervention was delivered by a clinical psychologist/neuropsychologist (first author with 8 years experience in mTBI), however, future application of ACTion mTBI will be delivered by other registered psychologists working in mTBI rehabilitation. |
| 1. **How**   Describe the modes of delivery (e.g., face-to-face or by some other mechanism, such as the internet or telephone) of the intervention and whether it was provided individually or in a group. | The intervention involved individual face-to-face sessions. Each session occurred predominately weekly. |
| 1. **Where**   Describe the type(s) of location(s) where the intervention occurred, including any necessary infrastructure or relevant features. | The intervention was delivered in a community mTBI rehabilitation service (called concussion services) located in Wellington, New Zealand. Concussion services in New Zealand are funded by Accident Compensation Cooperation (ACC), a government funded no-fault insurance scheme. |
| 1. **When and how much**   Describe the number of times the intervention was delivered and over what period of time including the number of sessions, their schedule, and their duration, intensity or dose. | The intervention consisted of 5 sessions (each 50 minutes in duration). Factors that affected a participant’s ability to receive the full dose of the intervention included participant illness, location problems, and if need for ongoing treatment for mental distress is needed. |
| 1. **Tailoring**   If the intervention was planned to be personalized, titrated or adapted, then describe what, why, when, and how. | A recent report by the Association for Contextual Behavioral Science (ACBS) task force (Hayes et al., 2021) stated: “It is unhelpful to allow applied psychological science to remain at the level of extensive intervention protocols, when the spirit of ACBS idiographic functional analysis linked to processes of change requires a more personalized approach” (pp. 176). In accordance with this recommendation, ACTion-mTBI was delivered with flexibility based on clinical judgment according to the participant's presentation. For example, if a central feature was severe fusion with unhelpful cognitions driving the presenting issues, then session 3 ‘unhooking from the mind’ was delivered earlier in the intervention. What is critical in the delivery of ACTion-mTBI is that the therapist adopts an ACT stance, using the ACTion-mTBI manual to guide delivery of the intervention and that all aspects of the ACT model are covered within the five sessions. |
| 1. **Modifications**   If the intervention was modified during the course of the study, describe the changes (what, why, when, and how). | No modifications were made to the intervention during the course of this study. |
| 1. **How well Planned:**   If intervention adherence or fidelity was assessed, describe how and by whom, and if any strategies were used to maintain or improve fidelity, describe them. | Fidelity checks of the ACTion-mTBI delivery were completed on 10% of sessions to ensure adherence to the ACT model, using the ACT fidelity checklist measure. This was done by a consultant clinical psychologist with extensive experience and training in ACT and mTBI. |
| 1. **How well Actual**:   If intervention adherence or fidelity was assessed, describe the extent to which the intervention was delivered as planned. | The average rating on the ACT-M was 34.0 out of a total score of 36 indicating high consistency with the ACT model. |
